# Integrated longitudinal metabolic monitoring before, during and after teplizumab in an adult with stage 2 type 1 diabetes: a case report

**DOI:** 10.3389/fendo.2026.1985205

**Published:** 2026-10-26

**Authors:** Agostino Milluzzo, Teresa Ballirò, Francesco Di Giacomo Barbagallo, Giosiana Bosco, Maurizio Di Marco, Giuseppe Frazzetto, Lucia Frittitta

**Affiliations:** 1 Department of Clinical and Experimental Medicine, University of Catania, Catania, Italy; 2 Diabetes and Obesity Center, Garibaldi-Nesima Hospital, Catania, Italy; 3 Department of Medicine and Surgery, “Kore” University of Enna, Enna, Italy; 4 Internal Medicine, Garibaldi-Nesima Hospital, Catania, Italy

**Keywords:** case report, continuous glucose monitoring, disease-modifying therapy, stage 2 type 1 diabetes, teplizumab

## Abstract

**Background:**

Teplizumab is the first disease-modifying therapy approved to delay progression from stage 2 to stage 3 type 1 diabetes (T1D). Although its efficacy has been demonstrated in randomized clinical trials, the optimal strategy for monitoring the early metabolic response after treatment has not yet been established.

**Case presentation:**

We report the case of a 19-year-old woman diagnosed with stage 2 T1D after severe corticosteroid-associated hyperglycemia unmasked an underlying autoimmune dysglycemic state. The diagnosis was confirmed by progressive deterioration in serial oral glucose tolerance test (OGTT), positivity for multiple islet autoantibodies and preserved endogenous insulin secretion. Following a standard 14-day course of intravenous teplizumab, the patient underwent prospective metabolic evaluation using serial OGTT, mixed-meal tolerance testing (MMTT), continuous glucose monitoring (CGM), pancreatic autoantibodies, screening for extra-pancreatic autoimmunity and safety assessments. Twelve weeks after treatment, OGTT normalized and stimulated C-peptide secretion remained preserved during MMTT, while CGM provided longitudinal characterization of ambulatory glucose profiles throughout the 24-week follow-up, without evidence of progression to stage 3 T1D.

**Conclusions:**

This case illustrates how OGTT, MMTT and CGM can provide complementary information on glucose tolerance, endogenous β-cell function and real-world glycemic homeostasis during early follow-up after teplizumab. Although no conclusions regarding optimal monitoring strategies can be drawn from a single patient, this longitudinal characterization may help inform the design of prospective studies evaluating metabolic monitoring after disease-modifying therapy in stage 2 T1D.

## Introduction

Type 1 diabetes (T1D) is a chronic autoimmune disease characterized by progressive immune-mediated destruction of pancreatic β-cells. The current staging model of T1D recognizes a prolonged presymptomatic phase during which individuals develop persistent islet autoimmunity before progressing to dysglycemia and, ultimately, overt diabetes. Stage 2 T1D, defined by the presence of two or more islet autoantibodies together with dysglycemia in the absence of clinical diabetes, represents a critical therapeutic window for interventions aimed at preserving residual β-cell function and delaying disease progression.

Teplizumab, a humanized Fc receptor non-binding anti-CD3 monoclonal antibody, is the first disease-modifying therapy shown to delay progression from stage 2 to stage 3 T1D. Herold et al. demonstrated that a single 14-day treatment course with teplizumab significantly prolonged the median time to clinical diabetes, confirming that immune intervention can modify the natural history of presymptomatic disease (1). Subsequent real-world studies have established the feasibility of teplizumab administration in routine clinical practice while providing initial evidence of early metabolic improvement and acceptable safety (2–7).

Despite these advances, the optimal strategy for monitoring the early metabolic response after treatment remains poorly defined. Published studies have mainly focused on delay of progression to stage 3 T1D, changes in oral glucose tolerance test (OGTT)-derived glucose values, glycated hemoglobin (HbA1c) or selected CGM metrics. Although each of these assessments provides clinically relevant information, none alone fully characterizes the complex metabolic phenotype of stage 2 T1D during and after immune intervention. The potential value of integrating dynamic glucose testing, physiological assessment of β-cell function and continuous glucose monitoring has not been systematically explored.

Here, we describe the case of a 19-year-old woman with stage 2 T1D prospectively evaluated before and after teplizumab using serial OGTT, mixed-meal tolerance testing (MMTT), continuous glucose monitoring (CGM), longitudinal pancreatic autoantibody assessment and, given the increased risk of associated autoimmune diseases in T1D, systematic screening for extra-pancreatic autoimmunity, together with treatment safety monitoring (8). Rather than focusing solely on the therapeutic response, the distinctive feature of this case is the integrated longitudinal metabolic characterization before, during and after teplizumab treatment, combining serial OGTT, MMTT-derived assessment of stimulated C-peptide secretion and extended CGM within the same patient.

## Case description

A 19-year-old Caucasian woman was referred to our Diabetes and Obesity Centre after an episode of severe hyperglycemia that occurred during a short course of oral betamethasone (2 mg/day for 5 days) prescribed for a dental abscess. One day after corticosteroid discontinuation, capillary blood glucose was 467 mg/dL. She presented to the Emergency Department, where 6 IU of a rapid-acting insulin analogue were administered, resulting in a reduction of plasma glucose to 121 mg/dL.

She denied previous episodes of hyperglycemia, polyuria, polydipsia, weight loss or diabetic ketoacidosis. Her family history was notable for diabetes mellitus in her maternal grandfather, who developed diabetes in his thirties and subsequently required insulin therapy. There was no family history of autoimmune disease. Past medical history was otherwise unremarkable except for previous patellar realignment surgery following traumatic injury.

Physical examination revealed a body weight of 60 kg, height of 163 cm and body mass index (BMI) of 22.6 kg/m². Initial laboratory evaluation showed a HbA1c of 5.5% (37 mmol/mol), indicating preserved average glycemic control despite the recent episode of marked hyperglycemia.

### Diagnostic assessment

A first OGTT, performed in early December 2025, showed plasma glucose concentrations of 102, 186 and 121 mg/dL at fasting, 60 and 120 minutes, respectively. Six weeks later, a repeat OGTT demonstrated the progression of dysglycemia, with fasting glucose increasing to 116 mg/dL, 1-hour glucose to 245 mg/dL and 2-hour glucose to 165 mg/dL, fulfilling criteria for impaired fasting glucose and impaired glucose tolerance (**Table 1**; **Figure 1a**). During the same period, fasting C-peptide declined from 2.20 to 1.45 ng/mL (**Table 1**; **Figure 1b**).

**Table 1:** Serial immunological and metabolic assessment before and after teplizumab treatment.

Variable (reference value)	Initial	Pre-treatment	12 weeks	24 weeks
Pancreatic autoimmunity				
GADA (<5 U/mL)	32.5	32.4	9.8	NA
IA-2A (<7.5 U/mL)	37.6	>4000	>4000	NA
ZnT8A (<15 U/mL)	>2000	>2000	>2000	NA
IAA (<10 U/mL)	NA	0.8	<0.1	NA
Extra-pancreatic autoimmunity				
TPO-Ab (<5.6 IU/mL)	NA	49.9	38.9	NA
Tg-Ab (<4.1 IU/mL)	NA	1.9	1.5	NA
TR-Ab (<1 U/mL)	NA	0.7	0.8	NA
tTG-Ab IgA	NA	negative	negative	NA
tTG-Ab IgG	NA	negative	negative	NA
PCA-Ab	NA	negative	negative	NA
Metabolic assessment				
HbA1c, % (mmol/mol)	5.5 (37)	5.5 (37)	5.3 (34)	NA
Fasting plasma glucose (mg/dL)	103	111	77	NA
OGTT glucose (0/60/120 min, mg/dL)	102/186/121	116/245/165	77/124/116	NA
Fasting C-peptide (ng/mL)	2.2	1.5	2.8	NA
Peak stimulated C-peptide during MMTT (ng/mL)	NA	NA	8.3	NA
Continuous glucose monitoring				
Mean glucose (mg/dL)	NA	119	109	99
GMI (%)	NA	6.2	5.9	5.7
CV (%)	NA	29.5	20.9	30.5
TIR (mg/dL, %) 70–140 70–180	NA NA	76 91	91 98	78 86
TAR (mg/dL, %) >140 >180	NA NA	22 7	9 2	10 2
TBR (<70 mg/dL, %)	NA	2	0	12

Initial, first diagnostic evaluation; Pre-treatment, assessment immediately before teplizumab administration; 3 months, comprehensive metabolic assessment performed 12 weeks after treatment; 24 weeks, CGM assessment performed 24 weeks after treatment completion.

GADA, glutamic acid decarboxylase antibodies; IA-2A islet antigen-2 antibodies; (ZnT8A), zinc transporter 8 antibodies; IAA insulin autoantibodies; TPO-Ab, antithyroid peroxidase antibodies; Tg-Ab, antithyroglobulin antibodies; TR-Ab, thyrotropin receptor antibodies; tTG-Ab, anti-transglutaminase antibodies; PCA-Ab, anti-parietal cell antibodies; HbA1c, glycated hemoglobin, OGTT, oral glucose tolerance test; MMTT, mixed-meal tolerance testing, GMI, glucose management indicator; CV, coefficient of variation; TIR, time in range; TAR, time above range; TBR, time below range; NA, not available.

**Figure 1:**
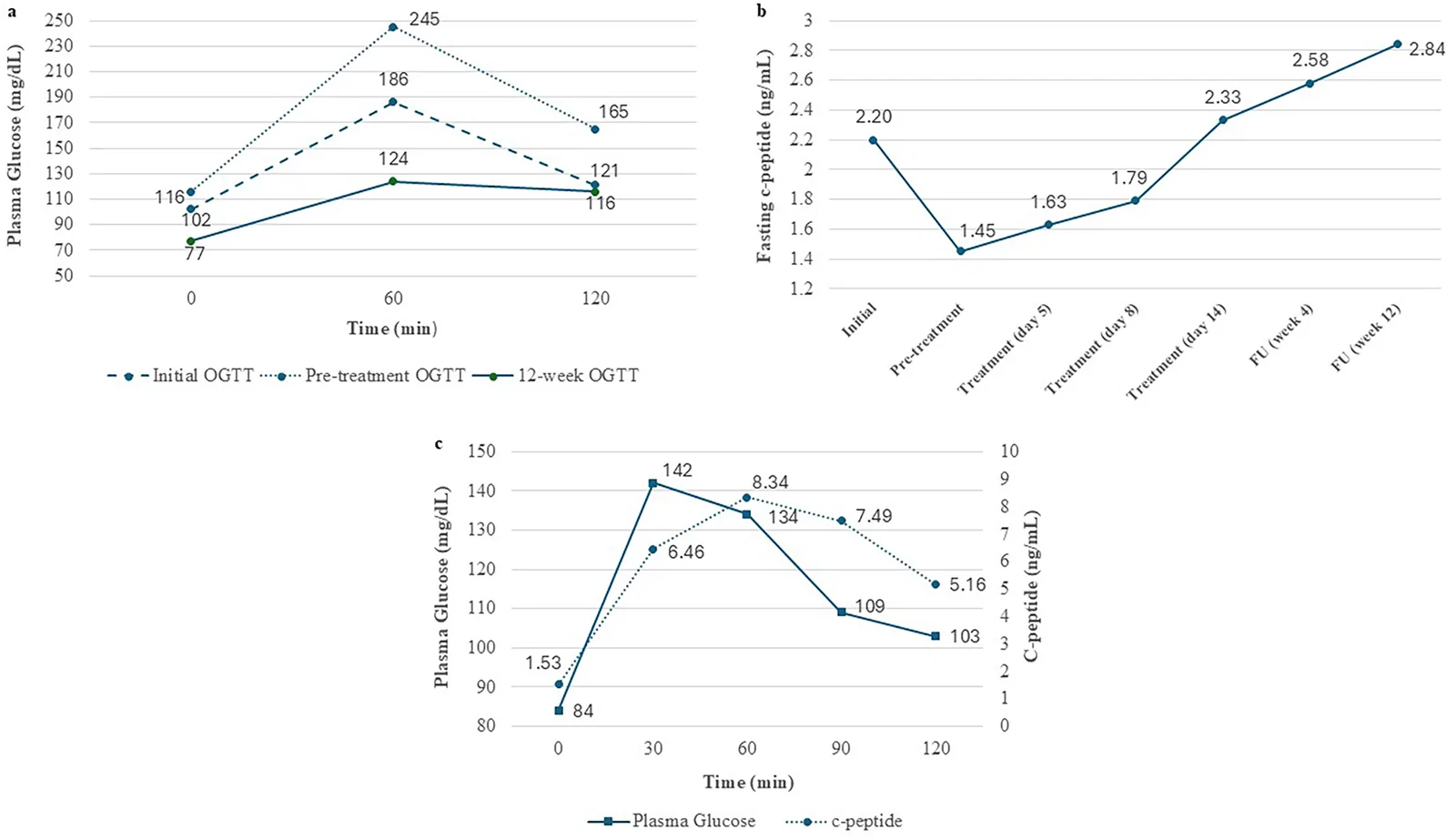
Longitudinal metabolic assessment before and after teplizumab treatment. **(a)** Plasma glucose concentrations during oral glucose tolerance tests (OGTTs) performed at the initial diagnostic evaluation, immediately before teplizumab treatment and at the 12-week follow-up. **(b)** Longitudinal changes in fasting C-peptide concentrations from the initial evaluation through teplizumab treatment and follow-up, including measurements obtained during treatment (days 5, 8 and 14) and at the 4 and 12-week follow-up visits. **(c)** Plasma glucose and C-peptide responses during the mixed-meal tolerance test (MMTT) performed 12 weeks after treatment.

Autoimmune assessment demonstrated triple positivity for glutamic acid decarboxylase autoantibodies (GADA), islet antigen-2 autoantibodies (IA-2A), and zinc transporter 8 autoantibodies (ZnT8A), whereas insulin autoantibodies (IAA) were negative. Comprehensive screening for associated autoimmune diseases was performed and included thyroid function, antithyroid peroxidase antibodies (TPO-Ab), antithyroglobulin antibodies (Tg-Ab), thyrotropin receptor antibodies (TR-Ab), anti-parietal cell antibodies (PCA-Ab), anti-transglutaminase antibodies (tTG-Ab) IgA and IgG, serum cortisol, sodium and potassium measurements. TPO-Ab were positive with normal TSH, free thyroxine and free triiodothyronine concentrations, consistent with euthyroid autoimmune thyroiditis. Screening for adrenal insufficiency, autoimmune gastritis and coeliac disease was negative (**Table 1**).

Based on the presence of dysglycemia together with positivity for three islet autoantibodies, the patient fulfilled diagnostic criteria for stage 2 T1D. Thus, before teplizumab administration, the patient had been characterized by serial OGTTs demonstrating progressive dysglycemia, declining fasting C-peptide, persistently normal HbA1c, and multiple islet autoantibody positivity, providing both metabolic and immunological characterization of stage 2 T1D at baseline.

Continuous glucose monitoring (CGM) was initiated four weeks before teplizumab administration, in late January 2026, using the FreeStyle Libre 2 Plus system (Abbott Diabetes Care, Alameda, CA, USA), and maintained throughout treatment and for 24 weeks after treatment completion to assess longitudinal ambulatory glucose profiles (**Figure 2**). The timing of metabolic assessments was selected to characterize complementary phases of the clinical course and was informed by available post-teplizumab follow-up data. Serial pre-treatment OGTTs were used to confirm persistent dysglycemia and characterize its evolution before therapy. A comprehensive metabolic reassessment was performed 12 weeks after treatment, consistent with the early follow-up window used in previous teplizumab studies, including OGTT to reassess glucose tolerance and MMTT to characterize stimulated endogenous β-cell function (2). CGM surveillance was subsequently continued through week 24 to extend the assessment of ambulatory glucose patterns beyond the 12-weeks metabolic evaluation. The overall clinical course, including the main diagnostic, therapeutic and follow-up milestones, is summarized in **Table 2**.

**Figure 2:**
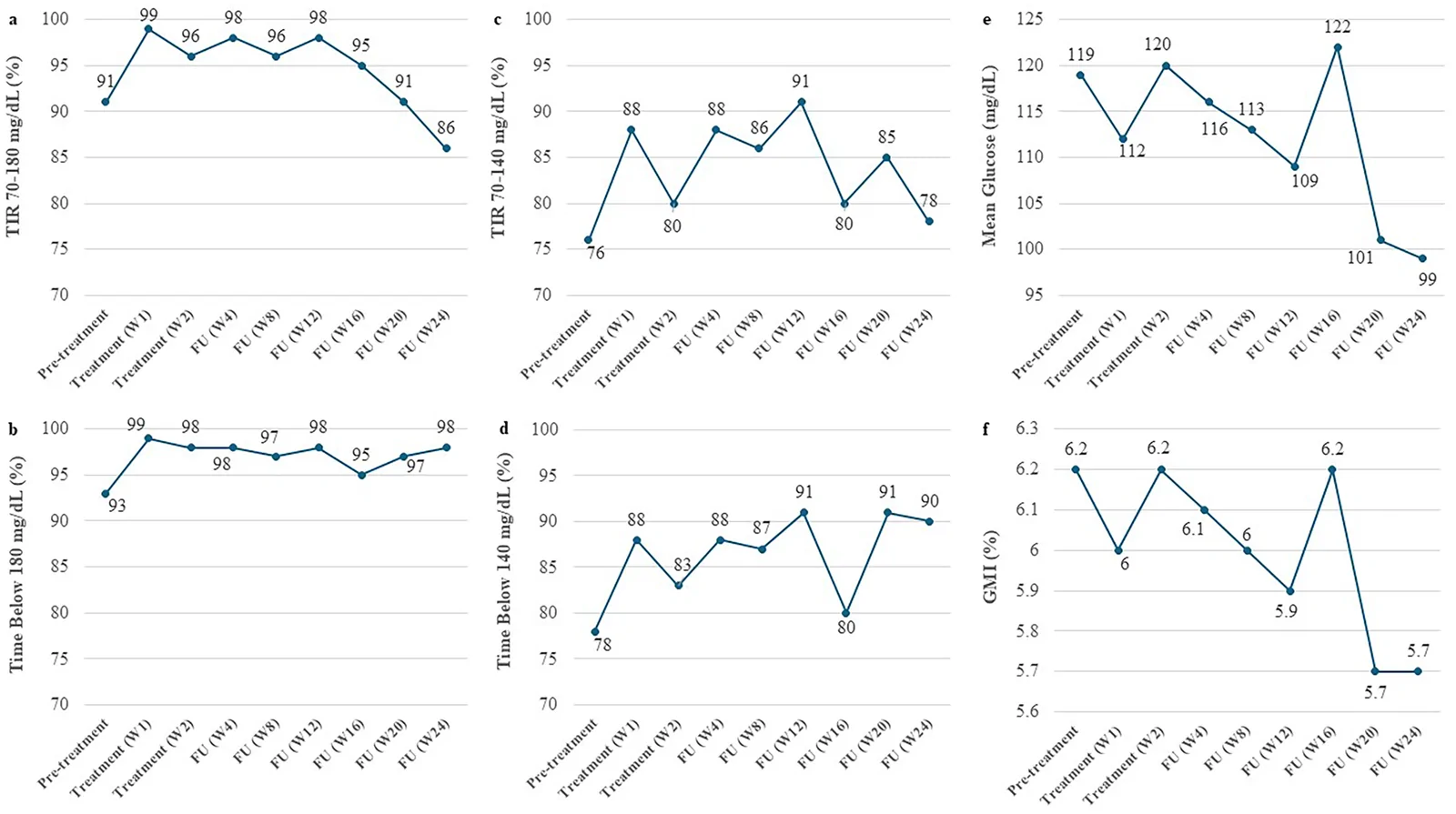
Longitudinal continuous glucose monitoring (CGM) metrics before, during and after teplizumab treatment. Panels show: **(a)** time in range (TIR) 70–180 mg/dL; **(b)** time below 180 mg/dL; **(c)** time in range 70–140 mg/dL; **(d)** time below 140 mg/dL; **(e)** mean glucose; and **(f)** glucose management indicator (GMI). Treatment weeks (W1–W2) correspond to the 14-day teplizumab administration period. Follow-up assessments were performed at 4-week intervals from week 4 through week 24 after treatment completion.

**Table 2:** Timeline of the clinical course.

Time	Clinical event and assessment
Initial presentation	Severe corticosteroid-associated hyperglycemia following a short course of oral betamethasone; referral for further metabolic assessment.
Early December 2025	Initial OGTT showed dysglycemia; fasting C-peptide 2.20 ng/mL.
6 weeks later	Repeat OGTT showed progression of dysglycemia with fasting C-peptide declining to 1.45 ng/mL. Multiple islet autoantibody positivity established the diagnosis of stage 2 T1D.
Late January 2026 (4 weeks before treatment)	CGM initiated and subsequently maintained throughout treatment and follow-up.
23 February–8 March 2026 (Treatment days 1–14)	Intravenous teplizumab administered according to the 14-day dosing schedule; serial metabolic and safety monitoring performed.
4 weeks after treatment	Fasting glucose and C-peptide follow-up.
12 weeks after treatment	Comprehensive metabolic assessment with OGTT, MMTT, C-peptide, CGM and autoimmune assessment.
24 weeks after treatment	Extended CGM follow-up completed; no evidence of progression to stage 3 T1D.

OGTT, oral glucose tolerance test; T1D, type 1 diabetes; CGM, continuous glucose monitoring; MMTT, mixed-meal tolerance testing.

### Therapeutic intervention

Following multidisciplinary evaluation, teplizumab was initiated on 23 February 2026 through a compassionate use, as the drug had been authorized by the European Medicines Agency (EMA) but had not yet been made available in Italy by the Italian Medicines Agency (AIFA). Teplizumab was administered by intravenous infusion once daily for 14 consecutive days according to the approved body surface area-based dosing schedule: 65 μg/m² on day 1,125 μg/m² on day 2,250 μg/m² on day 3,500 μg/m² on day 4, and 1,030 μg/m² on days 5–14. A midline catheter was inserted before treatment initiation to facilitate the 14-day intravenous infusion protocol. No vascular access-related complications occurred.

Oral premedication consisted of paracetamol (1000 mg on day 1), ibuprofen (400 mg once daily from days 2–14), ebastine (10 mg once daily from days 1–14) and metoclopramide (10 mg once daily from days 1–8).

The full 14-day treatment course was completed as planned, without dose reductions, delays or treatment interruptions. Transient lymphopenia developed between treatment days 4 and 5, with a nadir absolute lymphocyte count of 600 cells/µL, followed by complete spontaneous recovery. Mild self-limiting thrombocytopenia (without platelet counts falling below the normal range), transient fluctuations of liver enzymes within the normal reference range, and mild bilateral zygomatic erythema without pain or pruritus were also observed. All events resolved spontaneously without specific intervention. No serious adverse events, cytokine release syndrome or treatment discontinuation occurred.

### Follow-up and outcomes

Throughout treatment, fasting plasma glucose remained stable (103–111 mg/dL), while fasting C-peptide progressively increased from 1.63 ng/mL on day 5 to 1.79 ng/mL on day 8 and 2.33 ng/mL on day 14, reaching 2.58 ng/mL and 2.84 ng/mL at the 4- and 12-week follow-up visits, respectively (**Table 1**; **Figure 1b**).

Twelve weeks after treatment, comprehensive metabolic assessment was performed. Follow-up OGTT demonstrated normalization of glucose tolerance, with fasting, 60-minute and 120-minute glucose values of 77, 124 and 116 mg/dL, respectively (**Table 1**; **Figure 1a**). Despite the normalization of OGTT and the improvement in CGM metrics, HbA1c remained essentially unchanged (5.3%, 34 mmol/mol), highlighting its limited sensitivity for detecting early metabolic changes in stage 2 T1D (**Table 1**). A standardized MMTT was performed after an overnight fast using Ensure Plus Advance (Abbott), administered at 6 mL/kg body weight. The liquid meal provided 150 kcal/100 mL, with energy derived from carbohydrates (45%), protein (24%), fat (29%) and fructo-oligosaccharides (FOS, 1%), according to the manufacturer’s nutritional composition. MMTT demonstrated preserved stimulated endogenous C-peptide secretion at 12 weeks, with plasma glucose concentrations of 84, 142, 134, 109 and 103 mg/dL and corresponding C-peptide concentrations of 1.53, 6.46, 8.34, 7.49 and 5.16 ng/mL at baseline, 30, 60, 90 and 120 minutes, respectively (**Table 1**; **Figure 1c**).

Longitudinal CGM analysis characterized ambulatory glucose profiles before treatment, throughout the 14-day teplizumab course and during the extended 24-week follow-up (**Figure 2**). Both TIR 70–180 mg/dL and the more stringent TIR 70–140 mg/dL showed favorable early changes during treatment and the first 12 weeks of follow-up, although some variability emerged thereafter. At week 24, TIR 70–180 mg/dL and TIR 70–140 mg/dL were 86% and 78%, respectively, TAR >140 mg/dL and >180 mg/dL were 10% and 2%, respectively, mean glucose was 99 mg/dL, GMI 5.7%, and CV 30.5%. At the same time point, sensor-derived TBR <70 mg/dL was 12%; however, the patient reported no symptoms suggestive of hypoglycemia, and these low-glucose readings were not confirmed by capillary or plasma glucose measurements. Overall, CGM showed stable ambulatory glucose profiles throughout the 24-week follow-up, without evidence of progression to stage 3 T1D (**Figure 2**).

Serial assessment of pancreatic autoantibodies showed heterogeneous changes, with a marked decline in GADA titers, persistently elevated ZnT8A levels, a substantial increase in IA-2A titers and persistently negative IAA (**Table 1**).

## Discussion

This case illustrates the complementary information provided by serial OGTT, MMTT and CGM during early metabolic follow-up after teplizumab in stage 2 T1D. Rather than evaluating the same physiological dimension, these assessments characterize distinct aspects of glucose tolerance, endogenous β-cell function and real-world glycemic homeostasis. Their combined use in this patient provided a detailed longitudinal metabolic characterization that could not have been obtained from any individual assessment alone.

Teplizumab is the first disease-modifying therapy shown to delay progression from stage 2 to stage 3 T1D (1), and recent real-world studies have confirmed its feasibility in routine clinical practice while reporting encouraging metabolic outcomes (2, 3, 5). However, published studies have mainly focused on progression to stage 3 diabetes or on selected metabolic endpoints, including OGTT-derived glucose values, HbA1c or CGM metrics, whereas relatively little research has addressed the integration of complementary metabolic assessments during early post-treatment follow-up. Compared with previous reports, this case provides a detailed prospective metabolic characterization of the early post-treatment course, integrating serial OGTT, physiological β-cell assessment by MMTT, continuous CGM, longitudinal pancreatic autoantibody profiling and systematic screening for associated autoimmune diseases within the same patient.

The severe hyperglycemic episode at presentation occurred in temporal association with corticosteroid exposure and was therefore likely precipitated or amplified by glucocorticoid-induced insulin resistance. However, isolated corticosteroid-induced hyperglycemia could not account for the subsequent findings. Importantly, the first OGTT was performed approximately eight weeks after corticosteroid discontinuation, when the acute metabolic effects of the short glucocorticoid course would no longer be expected to persist. Serial OGTTs subsequently demonstrated persistent and progressive dysglycemia, accompanied by a decline in fasting C-peptide despite persistently normal HbA1c, and positivity for three islet autoantibodies. Thus, corticosteroid exposure was interpreted as a metabolic stressor that unmasked an underlying autoimmune dysglycemic state rather than as the sole cause of the glucose abnormality.

Twelve weeks after teplizumab, OGTT normalized and MMTT demonstrated preserved stimulated C-peptide secretion. However, the absence of a pre-treatment MMTT precludes assessment of longitudinal changes in stimulated β-cell function. The 12-week MMTT nevertheless provides a reference for comparison with subsequent MMTTs during longitudinal follow-up. CGM documented ambulatory glucose patterns throughout the extended 24-week follow-up. Although spontaneous variability of OGTT cannot be excluded in an individual patient, the concordance between standardized glucose testing, stimulated C-peptide secretion and ambulatory glucose profiles could support a coherent early metabolic trajectory. However, spontaneous fluctuations in glucose tolerance and β-cell function may occur during the natural history of stage 2 T1D, and a transient spontaneous metabolic improvement or remission-like fluctuation cannot be excluded. This should not be equated with the classical ‘honeymoon’ phase typically described after clinical diagnosis of stage 3 T1D and initiation of insulin therapy. Therefore, in this single uncontrolled case, the contribution of teplizumab cannot be separated from the natural variability of the underlying disease.

Importantly, each metabolic assessment contributed unique and complementary information. OGTT remained the reference method for evaluating changes in glucose tolerance, whereas MMTT provided a physiological assessment of endogenous β-cell function under nutrient stimulation. Continuous glucose monitoring complemented these dynamic tests by documenting glycemic behavior during everyday life, capturing changes in glucose exposure and variability that cannot be inferred from standardized testing alone. In addition, extending CGM surveillance to 24 weeks allowed assessment of the persistence and variability of ambulatory glucose patterns beyond the 12-week structured metabolic assessment. Notably, the sensor-derived TBR <70 mg/dL increased to 12% at week 24 despite the absence of reported hypoglycemic symptoms and glucose-lowering therapy. This finding should be interpreted cautiously, as previous studies have reported reduced FreeStyle Libre 2 accuracy in the low-glucose range, including underestimation of capillary glucose in healthy adults (9). Thus, sensor-related measurement variability may have contributed to the observed TBR; however, this possibility cannot be confirmed in the absence of simultaneous capillary or plasma glucose measurements.

Conversely, HbA1c remained essentially unchanged despite measurable improvements in both OGTT and CGM, reinforcing its limited sensitivity for detecting early metabolic changes in stage 2 T1D. Collectively, these findings indicate that the different metabolic approaches are not interchangeable but provide complementary information on distinct aspects of disease evolution.

Our observations are consistent with the largest prospective real-world experience reported to date. In the Barbara Davis Center cohort, treated individuals showed significant improvements in OGTT-derived glucose values during the first months after therapy together with favorable changes in HbA1c and CGM metrics (2). Similarly, Krepel-Volsky et al. demonstrated that CGM identifies heterogeneous metabolic trajectories after teplizumab, with increasing glycemic variability and glucose excursions characterizing the participant who progressed to stage 3 T1D despite relatively modest changes in HbA1c (6). Likewise, the Italian compassionate-use experiences reported by Guarnotta et al. and Conti et al. confirmed the feasibility and favorable short-term metabolic profile of teplizumab in adults (3, 5). Taken together, available evidence and the observations from this case indicate that OGTT, MMTT and CGM capture distinct aspects of glucose regulation and β-cell function. However, the present case does not establish the efficacy of teplizumab at the individual level nor validate a specific metabolic monitoring strategy. Whether the combined use of these assessments improves clinical monitoring after teplizumab requires evaluation in larger prospective cohorts.

Longitudinal pancreatic autoantibody assessment provided additional insights. Although GADA titers declined substantially after treatment, IA-2A and ZnT8A remained persistently elevated, whereas IAA remained negative, indicating that favorable metabolic evolution was not paralleled by a uniform reduction in humoral autoimmunity. This apparent dissociation is biologically plausible, since teplizumab primarily modulates autoreactive T-cell responses rather than antibody production. Accordingly, recent real-world studies have demonstrated stronger associations between metabolic improvement and changes in autoreactive T-cell populations than with conventional islet autoantibody titers (2), while Wilson et al. described improved glucose tolerance despite persistent autoimmune disease in patients with autoimmune polyendocrine syndrome type 1 (4). These findings suggest that pancreatic autoantibody titers remain valuable for disease characterization but should not be regarded as surrogate markers of early therapeutic efficacy.

The safety profile observed in our patient was fully consistent with previous clinical trials and real-world experiences, with transient lymphopenia representing the expected pharmacodynamic effect of anti-CD3 therapy and no serious treatment-related adverse events.

This case has several strengths, including the prospective observation of metabolic deterioration before treatment, uninterrupted CGM from the pre-treatment period through treatment and follow-up, serial OGTTs, fasting and stimulated C-peptide measurements, longitudinal pancreatic autoantibody assessment and comprehensive screening for associated autoimmune diseases. Several limitations should also be acknowledged. As a single case, this report cannot establish causality or distinguish a treatment-related effect from spontaneous metabolic and β-cell functional variability during the natural history of stage 2 T1D, nor can it predict long-term clinical benefit. Comprehensive metabolic assessment was performed at 12 weeks after treatment, whereas CGM surveillance was extended to 24 weeks. Although this provided information on the early post-treatment course, longer follow-up is required to determine whether the observed metabolic patterns are sustained over time and, importantly, whether progression to stage 3 T1D is delayed. Furthermore, a pre-treatment MMTT was unavailable, precluding direct comparison of stimulated β-cell function before and after therapy. In addition, the increased sensor-derived TBR <70 mg/dL observed at week 24 was not associated with reported hypoglycemic symptoms and was not confirmed by capillary or plasma glucose measurements, limiting its interpretation.

In conclusion, this case provides a detailed longitudinal characterization of metabolic changes before, during and after teplizumab treatment in an adult with stage 2 T1D. OGTT, MMTT and CGM provided complementary information on glucose tolerance, endogenous β-cell function and ambulatory glucose patterns, illustrating how different metabolic assessments can characterize complementary aspects of the post-treatment metabolic course. These observations are hypothesis-generating and should not be interpreted as evidence for a specific monitoring strategy. Larger prospective studies are needed to determine whether integrated metabolic assessment can improve the evaluation of treatment response and disease progression after disease-modifying therapy.

## Patient perspective

The patient approached the diagnosis of stage 2 T1D with a calm and well-informed attitude, supported by her family and with awareness of the risk of progression to clinical diabetes. She was highly receptive to the opportunity to receive teplizumab after discussing its potential benefits in delaying disease progression and their possible implications for future quality of life. She also showed strong engagement with longitudinal metabolic monitoring and readily agreed to continuous glucose monitoring throughout treatment and follow-up.

## Data Availability

The original contributions presented in the study are included in the article/supplementary material. Further inquiries can be directed to the corresponding author/s.
